# A novel bio-inspired strategy to prevent amyloidogenesis and synaptic damage in Alzheimer’s disease

**DOI:** 10.1038/s41380-022-01745-x

**Published:** 2022-08-26

**Authors:** Marcella Catania, Laura Colombo, Stefano Sorrentino, Alfredo Cagnotto, Jacopo Lucchetti, Maria Chiara Barbagallo, Ilaria Vannetiello, Elena Rita Vecchi, Monica Favagrossa, Massimo Costanza, Giorgio Giaccone, Mario Salmona, Fabrizio Tagliavini, Giuseppe Di Fede

**Affiliations:** 1 Neurology V – Neuropathology Unit, Fondazione IRCCS Istituto Neurologico Carlo Besta, Via Celoria 11, 20133 Milan, Italy; 2 Department of Molecular Biochemistry and Pharmacology, IRCCS Istituto di Ricerche Farmacologiche Mario Negri, Milan, Italy; 3 Molecular Neuro-Oncology Unit, Fondazione IRCCS Istituto Neurologico Carlo Besta, Via Celoria 11, 20133 Milan, Italy

**Keywords:** Neuroscience, Drug discovery, Molecular biology, Biochemistry, Biological techniques

## Abstract

Alzheimer’s disease (AD) is an irreversible neurodegenerative disorder that affects millions of people worldwide. AD pathogenesis is intricate. It primarily involves two main molecular players—amyloid-β (Aβ) and tau—which actually have an intrinsic trend to generate molecular assemblies that are toxic to neurons. Incomplete knowledge of the molecular mechanisms inducing the onset and sustaining the progression of the disease, as well as the lack of valid models to fully recapitulate the pathogenesis of human disease, have until now hampered the development of a successful therapy for AD. The overall experience with clinical trials with a number of potential drugs—including the recent outcomes of studies with monoclonal antibodies against Aβ—seems to indicate that Aβ-targeting is not effective if it is not accompanied by an efficient challenge of Aβ neurotoxic properties. We took advantage from the discovery of a naturally-occurring variant of Aβ (Aβ_A2V_) that has anti-amyloidogenic properties, and designed a novel *bio-inspired* strategy for AD based on the intranasal delivery of a six-mer peptide (Aβ1-6_A2V_) retaining the anti-amyloidogenic abilities of the full-length Aβ_A2V_ variant. This approach turned out to be effective in preventing the aggregation of wild type Aβ and averting the synaptic damage associated with amyloidogenesis in a mouse model of AD. The results of our preclinical studies inspired by a protective model already existing in nature, that is the human heterozygous Aβ_A2V_ carriers which seem to be protected from AD, open the way to an unprecedented and promising approach for the prevention of the disease in humans.

## Introduction

Alzheimer’s disease (AD) is the most common form of dementia and its prevalence is increasing with aging population [1]. This illness affects 6 million people in the EU and upwards of 30 million individuals worldwide. These figures are projected to increase substantially as the world population ages rapidly. AD is an irreversible neurodegenerative disease that causes disruptions in cognition, personality and other functions eventually leading to death from complete brain failure [2]. The impact on the quality of life of patients and their families is severe, accompanied by immense psychological pain. Thus, AD is a growing public health concern and the massive economic burden associated with this disease must be considered in the design of worldwide health programs. A central feature in the pathogenesis of AD is the intracerebral accumulation of neurotoxic forms of amyloid-β peptide (Aβ)—mostly small soluble oligomers—that is generated by the cleavage of the Amyloid Precursor Protein (APP) [3]. However, detailed knowledge of the molecular machinery involved in the illness has not yet been achieved [4, 5]. As a consequence, the unresolved complexity of this pathology has resulted in the failure of a huge number of clinical trials despite substantial efforts and robust investment in the development of disease-modifying compounds [6, 7]. No new drugs have been approved for AD during the past 17 years. The available medications have a very low impact on the disease course [8, 9]. Most clinical trials developed during the last decades have reached frustrating results even because the potential drugs were tested in the clinical phase of AD, i.e. years or even decades after the onset of the most relevant mechanistic events in the pathogenesis of the disease [7, 10]. Hence, a preventive rather than a curative approach against AD is hoped for now more than ever.

Sporadic AD accounts for more than 95% of all cases and is most probably the outcome of multiple genetic variants and unidentified environmental factors. Only a very small proportion of AD is caused by monogenetically inherited mutations [11]. In 2009, we described a genetic variant of the amyloid-β protein consisting of an alanine-to-valine substitution at codon 673 of the *APP* gene, corresponding to the second residue of Aβ sequence (Aβ_A2V_), that causes early-onset AD only in the homozygous state, while heterozygous carriers are unaffected even in advanced age [12]. The studies on molecular mechanisms responsible for the protective effects of the A2V mutation in heterozygous carriers revealed the exciting ability of the Aβ_A2V_ variant to hinder amyloidogenesis [13–15]. X-ray and neutron diffraction experiments combined with polarized light microscopy, atomic force microscopy (AFM) and modelling provided a rational basis for the paradoxical effects of A2V mutation, that is disease aggressiveness in homozygous carriers and protection in heterozygous carriers. Since the N-terminal Aβ residues are involved in inter-fibrillar interactions [16] and metal coordination [17], it is conceivable that valine-valine interactions in A2V homozygous carriers favor fibril polymerization and interaction with adjoining fibrils, as compared to alanine-alanine interaction in non-mutated subjects. Conversely, a mismatch in the packing likely occurs in A2V heterozygous carriers, due to unfavourable alanine-valine interaction, disrupting the hydrogen-bonding and inter-sheet organization, and thereby preventing fibrillogenesis [13]. A protective effect against AD and aging-related cognitive decline was claimed in the Icelander population for another Aβ variant (Aβ_A2T_) consisting of an alanine-to-threonine substitution at the same codon of the A2V mutation [18]. The discovery of *protective* genetic variants such as Aβ_A2V_ and Aβ_A2T_ opens a new way for preventing AD [19–22]. Distinct from previous attempts based on pure theoretical grounds, this strategy stems from the clinical observations that naturally-occurring Aβ variants actually offer protection against the disease.

Following this approach, we carried out in vitro studies with an *all-D-isomer* synthetic peptide limited to the first six amino acids of the N-terminal sequence of the A2V-mutated Aβ [Aβ1-6_A2V_ (D)]. This short peptide displayed an extraordinary ability to interact with wild-type full-length Aβ, interfering with its nucleation or nucleation-dependent polymerization [12]. We also showed that this peptide, conjugated with the short amino acid sequence derived from the HIV TAT peptide—widely used for brain drug delivery [23]—(a) inhibits oligomer generation, fibril formation and amyloid accumulation [14]; (b) reverses the synaptopathy induced by Aβ in hippocampal neurons [24]; (c) protects transgenic *C. elegans* from Aβ-induced neuromuscular damage [25]. Moreover, a previous attempt to prevent in vivo amyloidogenesis, Aβ-dependent neurotoxicity and synaptic dysfunctions in two mouse models of AD using Aβ1-6_A2V_-TAT(D) resulted in a successful outcome in short term treatment schedules. Unfortunately, more prolonged treatment schedules (5 months) with the Aβ1-6_A2V_-TAT(D) compound, although retaining the results on the prevention of cognitive impairment, attenuated the effects on Aβ production and paradoxically increased amyloid burden, most likely due to the intrinsic amyloidogenic properties of TAT carrier [26, 27] that neutralized the anti-amyloidogenic ability of the Aβ_A2V_ variant [19].

Here we describe a novel approach based on intranasal brain delivery of the Aβ1-6_A2V_ peptide alone—without any carrier. This strategy was designed to avoid potential TAT-like side effects or undesired counteractions on the primary relevant properties of Aβ1-6_A2V_ and led to an efficient in vivo prevention of Aβ oligomerization and synaptic damage in a double transgenic mouse model of AD.

Our results further support the hypothesis that AD can be prevented by using *bio-inspired* strategies based on drug compounds developed from protective genetic variants of amyloid–β protein.

## Materials and methods

### Peptide synthesis

The target peptide, corresponding to the N-terminal sequence of β-amyloid 1-42_A2V_, was synthesized on an automated Alstra synthesizer (Biotage, Uppsala, Sweden) at 0.1 mM scale with NOVASYN-TGA resin (Novabiochem, Darmstadt, Germany) using Fmoc-protected D-amino acids (Sigma Aldrich, Laufelfingen, CH). Amino acids were activated by a reaction with *O*-(Benzotriazole-1-yl)-*N*,*N*,*N’*, *N’*-tetramethyluronium tetrafluoroborate, and *N*, *N*-diisopropylethylamine. A capping step with acetic anhydride after the last coupling cycle of each amino acid was included. The peptide was cleaved from the resin with trifluoroacetic acid/thioanisole/water/phenol/ethanedithiol (82.5:5:5:5:2.5 vol/vol), precipitated, and washed with diethyl ether. The precipitate was purified by reverse-phase high-performance liquid chromatography on a semi-preparative C18 column (Waters Corporation, Milford, MA). The proper peak fraction corresponding to peptide molecular weight was identified using a MALDI-TOF spectrometer (Applied Biosystems, Concord, Ontario, Canada), freeze-dried, and stored at −20 °C until use. The peptide purity was higher than 95% [13].

### Animals

Animal care and experimental procedures involving animals were conducted in accordance with European Union (2010/63/EU) and Italian (D. Lgs. 26/2014) legislations and followed the applicable rules and guidelines of the institutional Animal care surveillance Committee. All the experiments were approved by the Animal care surveillance Committee of Carlo Besta Neurological Institute and by the Italian Ministry of Health.

We used 17 weeks old female APPSwe/PS1dE9 mice—strain B6;C3-Tg(APPswe,PSEN1dE9)85Dbo/Mmjax—expressing a chimeric mouse/human APP carrying the Swedish mutation and a human PS1 carrying the dE9 mutation. The animals were treated with PBS (*n* = 10) or 50 mg/kg Aβ1-6_A2V_(D) (*n* = 10) every 48 h for 20 weeks by intranasal administration, according to a previously published protocol [28]. A simple randomization was used to allocate animals to the experimental groups. 48 h after the last administration the mice were sacrificed and the brain was removed. The right hemibrain was fixed in 10% formalin for immunohistochemical analysis, and the left hemibrain was snap-frozen in liquid nitrogen and stored at −80 °C, after dissection of the hippocampus, for measurement of Aβ levels and analysis of APP processing. The hippocampus was used for the assessment of synaptopathy. The investigators were blinded during the assessment of the results.

### MALDI-imaging studies

Preliminary studies were carried out to confirm that the intranasal administration of Aβ1-6_A2V_(D) bypasses the blood-brain-barrier (BBB) and allows the efficient distribution of the peptide in the brain tissue. To this end, mice were treated every 48 h for 4 weeks at a dose of 20 mg/kg with peptide dissolved in PBS and sacrificed 4, 24 or 48 h (*n* = 3 animals/timepoint) after the last administration. Brains were removed and frozen immediately in liquid nitrogen and stored at −80 °C before sectioning. Sagittal sections (14 μm thickness) were prepared at −20 °C in a cryostat, mounted on steel plate Matrix-Assisted Laser Desorption/Ionization (MALDI) targets (Opti-TOF High-Resolution T.I.S., Applied Biosystem, Concord, Ontario, Canada) using a small paintbrush, and subsequently placed under vacuum at 4 °C overnight and stored at −20 °C until use. On the day of the experiment, mounted tissue sections were coated with a matrix solution of α-Cyano-4-hydroxycinnamic acid (15 mg/ml) dissolved in 60% acetonitrile/0.2% trifluoroacetic acid using a glass nebulizer. The plate was dried at room temperature for some minutes and was finally inserted into a MALDI-TOF mass spectrometer (4800 MALDI-TOF, Applied Biosystem, Concord, Ontario, Canada). For correct quantification and brain distribution of the Aβ1-6_A2V_(D) peptide, the molecular weight of the peptide was used as an external standard for generating MALDI-TOF brain imaging.

### Assessment of levels of Aβ monomers and oligomers in brain tissue

The left hemisphere of each brain was homogenized in 7 volumes of 10 mM Tris-HCl, pH 7.4, added with cOmplete Mini Protease Inhibitors cocktail (Roche, Mannheim, Germany), sonicated for 1 min using an ultrasonic homogenizer (SONOPULS) and centrifuged at 100,000 x *g* for 1 h at 4 °C. The supernatant was saved as the soluble fraction; the pellet was re-homogenized in 10 mM Tris-HCl, pH 7.4, 2% SDS added with cOmplete Mini Protease Inhibitors cocktail (Roche, Mannheim, Germany), sonicated for 1 min and centrifuged at 100,000 x *g* for 1 h at 4 °C. The supernatant was saved as the membrane fraction and the pellet was extracted in 70% formic acid and neutralized with 20 volumes of 1 M Tris (insoluble fraction).

Aβ40, Aβ42 and aggregated Aβ were measured in both soluble and insoluble fractions by ELISA (Invitrogen, Vienna, Austria). Each experiment was performed in triplicate. The analysis of APP processing was performed on the membrane fraction by western blotting with the A8717 antibody (cat A8717, Sigma, Saint Louis, MO). The signal intensity of the bands was measured using Quantity One (BioRad).

### Neuropathological studies

Following previously described protocol [19], coronal slices of the right hemibrain collected from transgenic mice were embedded in paraffin and cut (7 μm); sections were then de-waxed in xylene, hydrated through serial alcohols to water, pre-treated with formic acid (80%) and incubated overnight with anti-Aβ antibody (cat 800701, 4G8, 1:4000; BioLegend, San Diego, CA). The signal related to the primary antibody was detected by using a biotinylated secondary antibody followed by horseradish streptavidin peroxidase and visualized with DAB. Amyloid deposition was quantified in brain tissue using Aβ immunostaining (4G8). The assessment of immunostaining intensity was conducted in two adjacent sections of the same brain area [29]. A parallel quantification of the Aβ plaque load was performed using image analysis software (NIS-elements-Nikon) [30, 31].

### Assessment of synaptopathy in hippocampal samples

The left hippocampus was fractionated slightly modifying a previously published protocol [32]. Briefly, the tissue was homogenized in ice-cold 0.32 M sucrose, 1 mM Hepes, 1 mM MgCl_2_, 1 mM EDTA, 1 mM NaHCO_3_, 0.1 mM PMSF at pH = 7.4 added with cOmplete Mini Protease Inhibitors cocktail (Roche, Mannheim, Germany) and phosphatases inhibitors (Sigma, Saint Louis, MO) and spun at 1000 x *g* for 10 min at 4 °C. The supernatant was collected and centrifuged at 16,000 x *g* for 15 min at 4 °C. The supernatant was discarded; the pellet, consisting of the membrane fraction, was homogenized in 75 mM KCl, 1% Triton X-100 added with protease and phosphatases inhibitors, incubated 10 min on ice and centrifuged at 100,000 x *g* for 1 h at 4 °C. The resulting pellet (Triton Insoluble Fraction) was homogenized in 20 mM Hepes added with protease and phosphatases inhibitors, sonicated and stored at −80 °C. Protein concentrations were measured with the BCA Protein Assay kit (Pierce, Rockford, IL). 5 μg of proteins were loaded into Bolt 4–12% Bis-Tris polyacrylamide gels (Invitrogen, Carlsbad, CA), transferred to nitrocellulose membranes and incubated with the following primary antibodies: NMDAR2A (cat PA5-35377, polyclonal,1:500, Invitrogen, Rockford, IL), NMDAR2B (cat MA1-2014, NR2B, 1:500, Invitrogen, Rockford, IL), GluR1 (cat MA5-27694, S355-1, 1:500, Invitrogen, Rockford, IL), GluR2 (cat AB1768-I, polyclonal, 1:500, Millipore, Temecula, CA), PSD-95 (cat MA1-045, 6G6-1C9, 1:2000, Invitrogen, Rockford, IL), alpha-tubulin (cat T9026, DM1A, 1:10000, Sigma, Saint Louis, MO). The membranes were then incubated with IRDye 680 or IRDye 800 secondary antibody (Li-Cor Biosciences, Lincoln, NE); the immunoreactive bands were visualized and quantified using the Odyssey Infrared Imaging System (Li-Cor Biosciences).

### Measurements of serum antibodies against Aβ peptides

Levels of Aβ peptides-specific IgG were detected as described [19]. In brief, 96-well plates (Immulon 4 HBX; Thermo Scientific, Thermo Scientific, Rochester, NY) were coated overnight at 4 °C with 0.1 ml of the peptide diluted in NaHCO3 buffer (pH 9.5, 0.1 M) at a concentration of 0.010 mg/ml. Unspecific binding was blocked with 3% milk dissolved in distilled water (blocking buffer) for 3 h at room temperature. Serum samples were diluted 1:40 in blocking buffer and incubated for 2 h at room temperature.  Antibody binding was revealed by incubation with a peroxidase-conjugated monoclonal goat anti-mouse IgG (Southern Biotechnology, Birmingham, AL) diluted 1:5000 in blocking buffer for 1 h at room temperature, followed by addition of 3,3’,5,5’-Tetramethylbenzidine (BD Bioscience, San Diego, CA). The plates were read at 450 nm on a microplate reader. Anti-Aβ1-42 IgG antibodies (clone 6E10, cat 803001, or 4G8, BioLegend, San Diego, CA) or sera from mice immunized with Aβ1-6_A2V_(D) emulsified in Complete Freund’s Adjuvant (CFA) were used as positive controls. Sera from naïve C57BL/6 mice were used as negative controls.

### Statistical analysis

The sample size was calculated by power analysis (1-β = 0.80, α = 0.05), based on our previous results obtained after the treatment of the same mouse model with the peptide Aβ1-6_A2V_(D) conjugated with the TAT peptide [19] and using the levels of Aβ42 and aggregated Aβ in soluble and insoluble fractions of brain homogenates.

A F-test was used to compare variances. Depending on the normality of the data and the equality of variances, the Mann Withney *U*-test or Student *t*-test were used to compare (i) amyloid burden in immunohistochemical studies, (ii) Aβ 40, Aβ 42 and aggregated Aβ levels obtained by ELISA tests, and (iii) relative amounts of APP C-terminal fragments after Western Blot quantification. A two tailed p value equal or less than 0.05 was considered statistically significant. All calculations were performed using GraphPad Prism 5.0 (Graph-Pad Software, La Jolla, CA). All data are presented as mean ± standard error of the mean.

## Results

### The intranasal delivery of Aβ1-6_A2V_(D) results in an efficient distribution of the peptide in the mouse brain

**Fig. 1:**
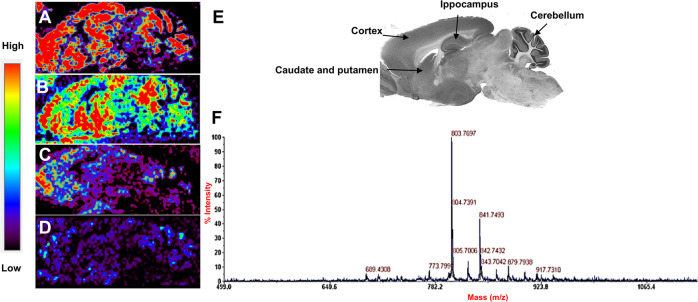
MALDI-TOF images of mouse brains distribution of the Aβ1-6_A2V_ peptide entirely composed of D-amino acids. Mice were sacrificed 4 (**A**), 24 (**B**) and 48 (**C**) hours after the last peptide administration. Control mice (**D**) were treated with saline solution. The lateral color bar indicates the peptide concentration in the different brain regions: blue, lower concentration; red, higher concentration. **E** shows a sagittal reference section from the Paxinos and Watson mouse brain atlas. The same peptide used for the mice treatment was used as a standard (**F**) for the correct peptide molecular weight identification in mouse brain areas.

A MALDI-TOF imaging study was carried out in a mouse brain to assess the efficacy of intranasal delivery to the brain of the Aβ1-6_A2V_(D) peptide. Figure 1 reports Aβ1-6_A2V_(D) distribution in the mouse brain after intranasal administration. The peptide was present in the main brain areas: the cerebral cortex, hippocampus, caudate-putamen and cerebellum. Notably, the peptide was still observed in the cerebral cortex 48 h after the last treatment. These observations supported the choice of a treatment schedule based on a 48 h interval between intranasal administrations of Aβ1-6_A2V_(D) to mice. We also quantified peptide levels in three different brain regions (Supplementary Fig. 1). In particular, the highest values were observed in the cortex and hippocampus 4 h after the treatment, and a slight decrease was observed at 24 h. At variance with these two areas, in the striatum the highest value was observed at 4 h and 24 h and declined at 48 h.

### Aβ1-6_A2V_(D) – based treatment inhibits Aβ aggregation in vivo

**Fig. 2:**
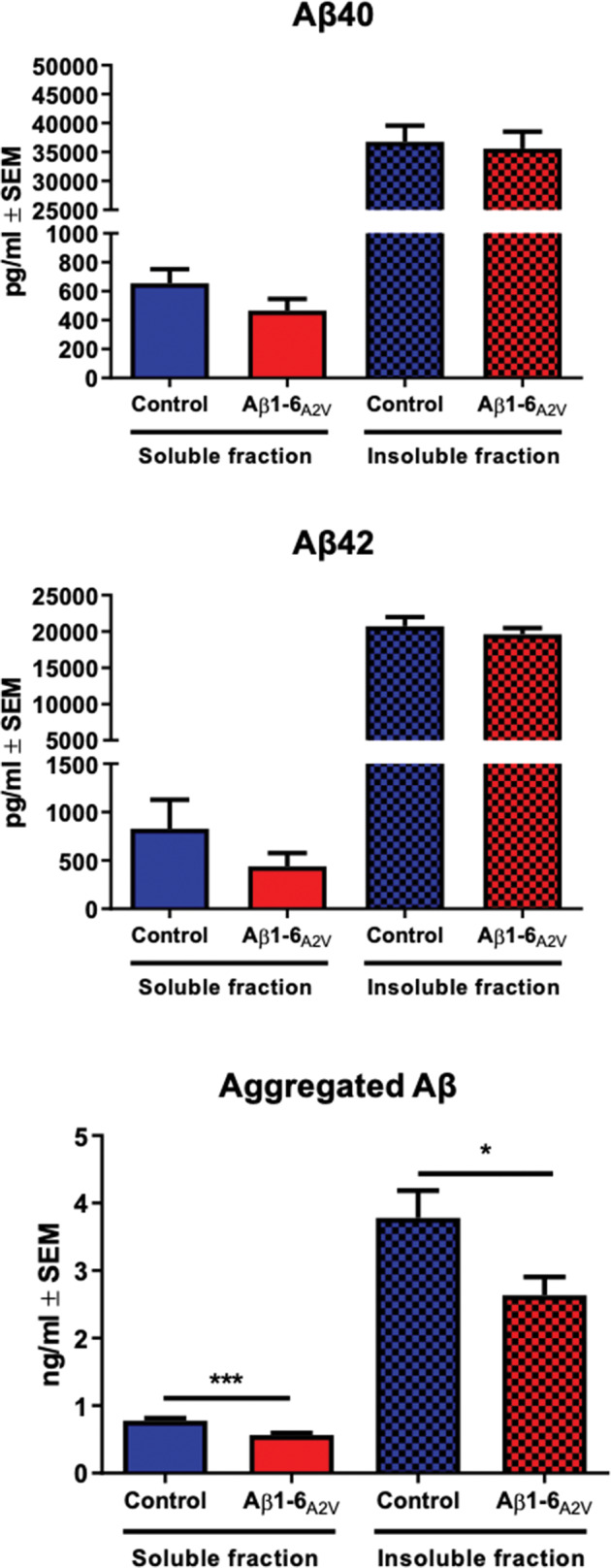
Effects of Aβ1-6_A2V_(D) on brain levels of Aβ monomers and oligomers. Biochemical study on APPSwe/PS1dE9 mice intranasally treated with Aβ1-6_A2V_(D) every 48 h for 5 months. Aβ40, Aβ42 and aggregated Aβ levels were measured by ELISA in soluble and insoluble fractions of brain homogenates from mice treated with saline solution (blue columns, control group, *n* = 10) or Aβ1-6_A2V_(D) (red columns, *n* = 10). Aggregated Aβ levels were significantly reduced in soluble (*** *p* = 0.0005) and insoluble (* *p* = 0.04) fractions of animals treated with Aβ1-6_A2V_(D) compared to controls.

The effects of Aβ1-6_A2V_(D) on Aβ production and polymerization were assessed by ELISA measuring Aβ40, Aβ42 and oligomeric Aβ in both soluble and insoluble fractions extracted from murine brain homogenates (Fig. 2). The mice treated with Aβ1-6_A2V_(D) did not show any significant difference in the levels of Aβ40 and Aβ42 as compared to controls. However, oligomeric Aβ levels showed a significant decrease in both soluble (*p* = 0.0005) and insoluble (*p* = 0.04) fractions, indicating that the intranasal administration of Aβ1-6_A2V_(D) is able to prevent the formation of neurotoxic Aβ aggregates in vivo. Moreover, the prolonged treatment with Aβ1-6_A2V_(D) did not substantially affect APP processing (Supplementary Fig. 2).

### Aβ1-6_A2V_(D) – based therapy prevents amyloid plaques formation

**Fig. 3:**
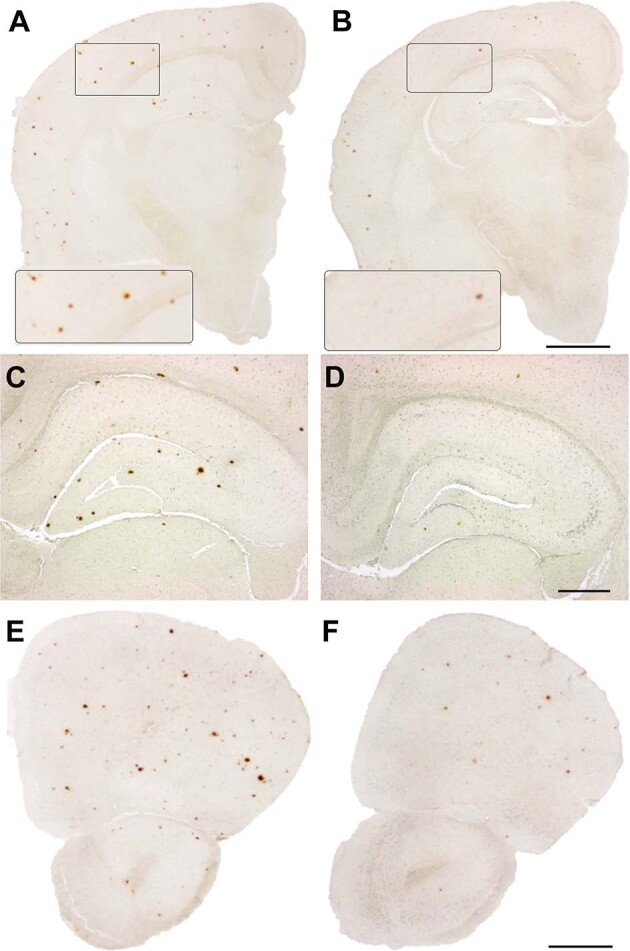
Effects of Aβ1-6_A2V_(D) on amyloid burden (neuropathological changes). Neuropathological study on APPSwe/PS1dE9 mice intranasally treated with Aβ1-6_A2V_(D) every 48 h for 5 months. Amyloid deposits in mice treated with Aβ1-6_A2V_(D) (**B**, **D**, **F**) vs mice treated with saline solution following the same treatment schedule (control group) (**A**, **C**, **E**). Immunohistochemistry with 4G8 antibody, original magnification 1x (**A**, **B**, **E**, **F**, scale bar = 1000 µm) and 4x (**C**, **D**, scale bar = 500 µm). Evidence of reduction of amyloid burden in several brain areas (cortex: **B** and insert; hippocampus: **D**; olfactory bulb: **F** of the group treated with Aβ1-6_A2V_(D) in comparison with the control group (cortex: **A** and insert; hippocampus: **C**; olfactory bulb: **E**.

**Fig. 4:**
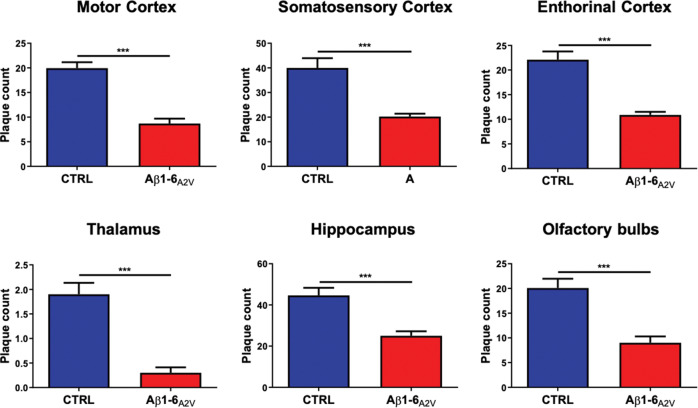
Effects of Aβ1-6_A2V_(D) on amyloid burden (densitometric analysis). Neuropathological study on mice intranasally treated with Aβ1-6_A2V_(D) every 48 h for 5 months Quantification of the effects of the treatment on amyloid burden was achieved by a densitometric analysis based on ‘plaque count’ in the two experimental groups: saline-treated animals (blue columns, *n* = 10) and Aβ1-6_A2V_(D)-treated mice (red columns, *n* = 10). Amyloid burden was strongly decreased in the group treated with Aβ1-6_A2V_(D) in comparison with mice treated with saline solution (control group) in several brain areas (motor cortex, somatosensory cortex, entorhinal cortex, thalamus, hippocampus, olfactory bulbs). The significance of the results was calculated using a Mann–Whitney *U* test. Statistical differences were considered significant if *p* < 0.05.

The effects of the treatment with Aβ1-6_A2V_(D) on Aβ deposition in brain tissue were analyzed by immunohistochemistry with the anti-Aβ 4G8 antibody. In comparison with the control animals (Fig. 3A, C, E), the intranasal administration of Aβ1-6_A2V_(D) efficiently prevented the formation of amyloid-β deposits which were much less represented in the brains of transgenic mice treated for 5 months (Fig. 3B, D, F). This effect was evident even in brain areas distant from the olfactory bulbs and always actively involved in the formation of amyloid plaques in the APPSwe/PS1dE9 model. The results of the immunohistochemical study were confirmed by the densitometric analysis of the amyloid burden in both the treated and control groups, with evidence of a strong reduction of Aβ deposition in the brains of mice treated with Aβ1-6_A2V_(D) through the intranasal route (motor cortex: *p* < 0.001; somatosensory cortex: *p* < 0.001; entorhinal cortex: *p* < 0.001; thalamus: *p* < 0.001; hippocampus: *p* < 0.001; olfactory bulbs: *p* < 0.001) (Fig. 4).

### Aβ1-6_A2V_(D) – based treatment preserves synaptic integrity

**Fig. 5:**
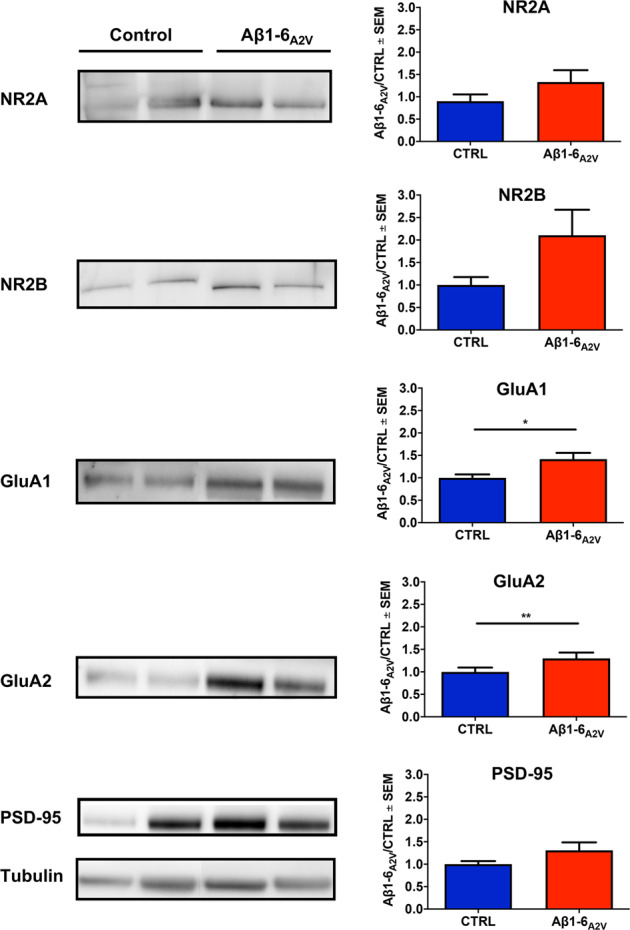
Effects of Aβ1-6_A2V_(D) on synaptic integrity. Biochemical study on APPSwe/PS1dE9 mice intranasally treated with Aβ1-6_A2V_(D) every 48 h for 5 months. The levels of the synaptic proteins NR2A, NR2B, GluA1, GluA2 and PSD-95 were analyzed by western blot. Two representative animals from each group are included in the figure. The densitometric analysis showed a significant increase in the levels of GluA1 (*p = 0.015) and GluA2 (**p = 0.007) and a trend toward an increase in the levels of NR2A, NR2B and PSD-95 in mice treated with Aβ1-6_A2V_(D) (red columns, *n* = 10) compared to animals treated with saline solution (blue columns, control group, *n* = 10).

The effects of the treatment with Aβ1-6_A2V_(D) on the synaptic damage associated with cerebral amyloidosis were assessed by analyzing the biochemical composition of the post-synaptic density obtained after sub-cellular fractionation of the left hippocampus. In particular, we analyzed the integrity of hippocampal synapses by measuring the levels of some AMPA and NMDA synaptic receptor subunits and of a scaffold synaptic protein. We found that the animals administered with Aβ1-6_A2V_(D) for 20 weeks showed significantly higher levels of GluA1 (*p* = 0.015) and GluA2 (*p* = 0.007) subunits of AMPA receptors compared to controls and a trend toward increasing levels of NR2A and NR2B subunits of NMDA receptors, as well as the scaffold protein PSD-95 (Fig. 5). These data suggest that prolonged treatment with Aβ1-6_A2V_(D) is able to prevent synaptic loss due to Aβ accumulation and oligomerization in the APPSwe/PS1dE9 transgenic model.

### Aβ1-6_A2V_(D) – based treatment does not result in an IgG response against Aβ1-6_A2V_ or Aβ1-42 peptides

To verify whether the administration of Aβ1-6_A2V_(D) provoked an antigen-specific immune response against either the therapeutic peptide or the full-length Aβ1-42, we measured serum titers of anti-Aβ1-6_A2V_(D) or anti-Aβ1-42 IgG in mice treated with Aβ1-6_A2V_(D) or PBS, before and 20 weeks after the start of the treatment. Of note, Aβ1-6_A2V_(D) did not induce detectable levels of IgG against Aβ1-6_A2V_(D) (Supplementary Fig. 3A) or Aβ1-42 (Supplementary Fig. 3B) at any of the time-points analyzed, suggesting that this treatment is endowed with poor immunogenicity.

## Discussion

AD has been in the line of fire with research on disease-modifying treatments for years. In 2020, at least 121 agents have been under evaluation in clinical trials for AD and many others are under investigation in preclinical studies [33]. Over the last few years, an increasing number of drug candidates against non-amyloid targets, such as anti-tau or anti-inflammatory drugs or compounds targeting synapses, vascular factors and neurogenesis have been proposed. However, until now all these approaches have failed to efficiently halt disease onset and progression. Even the most advanced and promising strategy based on the use of monoclonal antibodies designed to bind to β-amyloid were inefficient in providing the intended clinical benefits on cognitive and functional measures in AD patients. Recently, one anti-amyloid antibody (aducanumab) was approved under the accelerated approval pathway by FDA as new disease-modifying compound in AD. However, there is still uncertainty about the real efficacy and safety of aducanumab, even taking into account the previous attempts with other monoclonal antibodies, and both academic institutions and pharmaceutical companies are actively in search of innovative treatments [34, 35]. The reasons for the glaring failure of the therapeutic strategies against AD are most likely multiple and include, among others, (i) the rationale of the therapeutic approaches that in some cases are based on theoretical assumptions or—even if validated in animal models—are too weak to go all the way down the *bench-to-bedside* route [36, 37], (ii) the timing of therapeutic intervention—which should be very early along the natural history of the disease—(iii) the phenotypic variability of AD [38]—that deeply affects and diversifies the responsiveness to treatments—and (iv) the complexity of disease pathogenesis suggesting that more than one target should be locked by therapeutic strategies to be successful in tackling the illness [7, 39, 40]. Overall these observations suggest that the optimal therapeutic strategy against AD should engage multiple allied molecular mechanisms, demonstrating efficacy not only in interfering with the well-known players in AD pathogenesis—i.e., Aβ, tau, neuroinflammation and others—but also in hindering their neurotoxic effects.

In the recent past, genetic studies have discovered rare mutations with putative protective effects against AD providing an exciting background for the development of novel investigational compounds for the treatment of the disease [18, 20, 41–43]. In this context, a bioinspired approach based on a genetic variant already existing in nature and harboring multiple protective effects against the disease may offer a solid basis for the development of a successful therapeutic strategy. Following this approach, we have generated a novel compound that promises to replicate the natural protection occurring in the human heterozygous carriers of the Aβ_A2V_ variant [12, 14, 44–46].

The treatment of the APPSwe/PS1dE9 transgenic mouse model of AD with the Aβ1-6_A2V_(D) through the intranasal route allowed us to avoid the counteracting effects of the TAT carrier [19]. Indeed, unlike Aβ1-6_A2V_-TAT(D), Aβ1-6_A2V_(D) alone did not alter APP processing and retained the ability to reduce Aβ oligomers and amyloid burden, and preserve synaptic integrity even in a long-term treatment schedule.

Concerning the effects of Aβ1-6_A2V_(D) on synapses, extensive data from scientific literature support the relevance of AMPA receptors in the synaptopathy associated with AD [47]. Indeed, high concentrations of soluble oligomeric Aβ cause endocytosis and removal of AMPA receptors [48]. Further evidence from in vivo studies indicate that the disruption of PSD-95—a postsynaptic scaffold protein of excitatory synapses that binds to NMDA and AMPA receptors—is associated with cognitive and learning deficits [49]. Reduced expression of PSD-95 is a recurrent feature in brain tissue from AD subjects and murine models of AD [50]. It is also known that Aβ1-42 inhibits synaptic plasticity [51] by enhancing endocytosis of NMDARs with consequent reduction of NMDARs expression at the postsynaptic level [52]. NR2A and NR2B levels are indeed decreased in susceptible regions of the human AD brain, such as the hippocampus and the cortex [53]. Overall, these findings reflect the disruptive actions of soluble Aβ on synaptic plasticity in AD, provide keys to interpreting the effects of the Aβ1-6_A2V_(D)—based strategy on AD-related synaptopathy and support the use of PSD-95, AMPA and NMDA receptor levels as indicators of efficacy for therapeutic strategies against AD aimed at preserving synaptic integrity [54].

In our opinion, these data provide a solid rationale for the use of Aβ1-6_A2V_(D) in preventing amyloidogenesis and its deleterious effects on synaptic function and cognition in AD. The approach based on D-peptides is very promising because of their characteristics including high resistance to protease digestion, stability, and bioavailability [37], which make them optimal molecular prototypes for the development of drugs for the treatment of neurological disorders [55]. Actually, Aβ1-6_A2V_(D) may be included in the class of ‘amyloid β-targeted peptide inhibitors’, which have special properties, including high selectivity, low accumulation in tissues, low side-effects and toxicity, and different chemical and biological synthesis routes when compared with other compounds used for therapeutic purposes [36, 56, 57].

On the other hand, the intranasal administration route is increasingly being used as a noninvasive method to bypass the BBB for drug delivery of therapeutics in a number of neurological diseases, including AD [58, 59]. It is known that small fractions of nasally applied macromolecules may reach the brain directly via olfactory and trigeminal nerve components of the nasal mucosa or by bulk flow and diffusion within perineuronal channels, perivascular spaces, or lymphatic channels directly connected to brain tissue [60, 61] and appear to rapidly distribute within the brains of rodents and primates. In our study, intranasal delivery of Aβ1-6_A2V_(D) resulted in high concentration and good distribution of the six-mer peptide in brain tissue of APPSwe/PS1dE9 mice, providing grounds for a highly compliant treatment for AD in humans, similar to other therapeutics already used in clinical practice [62, 63].

In a nutshell, the Aβ_A2V_-based strategy has at least two main advantages compared to the previous therapeutic approaches for AD [64–67]. First, it stems from a “protective” model already existing in nature: APP-A673V heterozygous carriers which are protected from AD occurrence. Second, it engages combined mechanisms of action and results in multiple allied effects on AD pathogenesis, involving inhibition of oligomer generation, fibril formation and amyloid accumulation, and interference with Aβ-dependent neurotoxicity and synaptic dysfunction that may delay cognitive impairment in animal models.

Moreover, the use of Aβ1-6_A2V_ (D) promises to be less expensive than other pharmacological treatments for AD—such as monoclonal antibodies—and guarantees high compliance for AD patients if the treatment can be performed through the intranasal route.

Most importantly, the timing of the treatment with Aβ1-6_A2V_(D)—which in our preclinical study on APPSwe/PS1dE9 mice started in an early phase of their disease, when the first amyloid deposits appear in the brain—suggests that the bio-inspired Aβ_A2V_-based strategy may be either a preventive or a curative approach to AD. Further studies are needed to set the most efficient treatment schedule and to exclude potential side effects of Aβ1-6_A2V_(D) that in any case have not been observed in mice.

In conclusion, this study stems from the discovery of a protective genetic variant of β-amyloid offering grounds for the development of AD therapeutics. This bio-inspired approach provides, in our opinion, a novel compound for the prevention and/or cure of AD and, in a more general context, opens the way for innovative therapeutic strategies based on the identification of naturally occurring genetic variants having protective effects in humans [12, 18, 19, 68–71]. Such strategies should be aimed at replicating in AD patients the condition occurring in the human carriers who are protected against the disease to prevent or halt the progression of AD.

## Supplementary information

Supplementary Information
