# Supplementary material for: A novel bio-inspired strategy to prevent amyloidogenesis and synaptic damage in Alzheimer’s disease
Source: Mol Psychiatry. 2022 Aug 26;27(12):5227–34. doi: 10.1038/s41380-022-01745-x (PMC9763104; doi:10.1038/s41380-022-01745-x)
Supplement: Supplementary file 1 — Supplementary Information [file 41380_2022_1745_MOESM1_ESM.docx]

**Supplementary Figures**


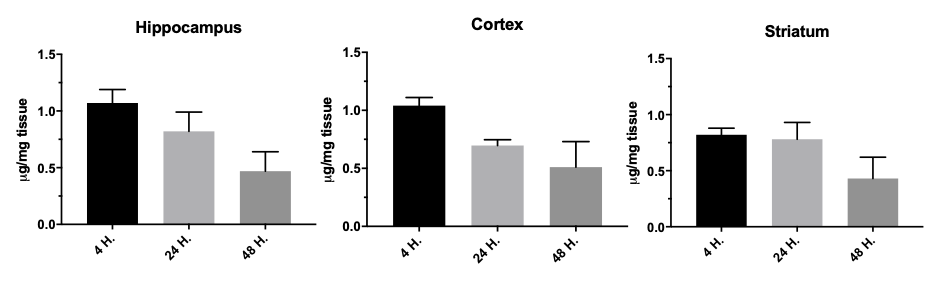


**A**

**B**

**Supplementary Fig. 1** Quantitative distribution of Aβ1-6_A2V_ (D) in different brain areas of mice treated with the peptide every 48 h for 4 weeks and sacrificed 4 h, 24 h and 48 h after the last intranasal administration. (**A**) Different peptide concentrations (from 0.1 to 2.0 µg/mg tissue) were spotted directly on a control tissue slide and used to generate a standard curve to evaluate peptide tissue levels in different brain regions. The ion signal increases linearly with the peptide amount. The linearity of the calibration curve was improved by normalization. (**B**) The peptide was found in the cerebral cortex, hippocampus, caudate-putamen, and cerebellum. The peptide was still detected in the brain tissue 48 h after the last treatment.


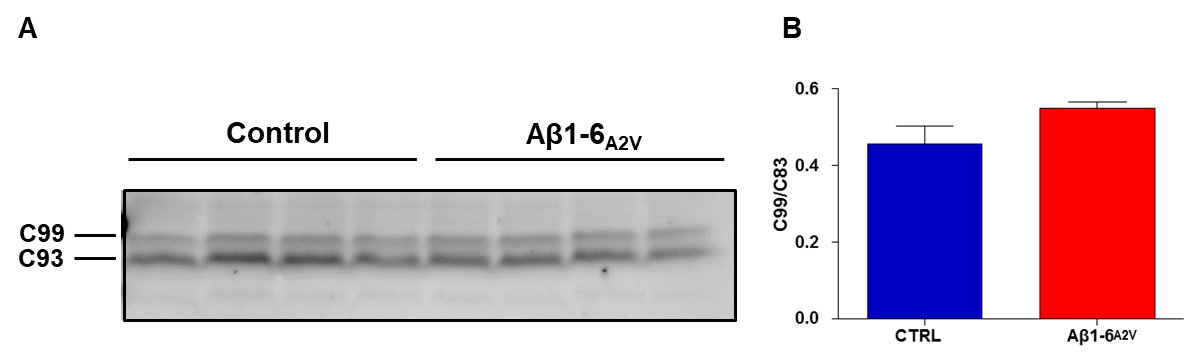


**Supplementary Fig. 2** Effects of Aβ1-6_A2V_ (D) on APP processing. (A) The levels of the amyloidogenic fragment C99 and the non-amyloidogenic fragment C83, derived from APP processing, were analyzed by western blot using the A8717 antibody. (B) Densitometric analysis did not show any significant difference in mice treated with the peptide (red, n = 10) compared with the controls (blue, n = 10).


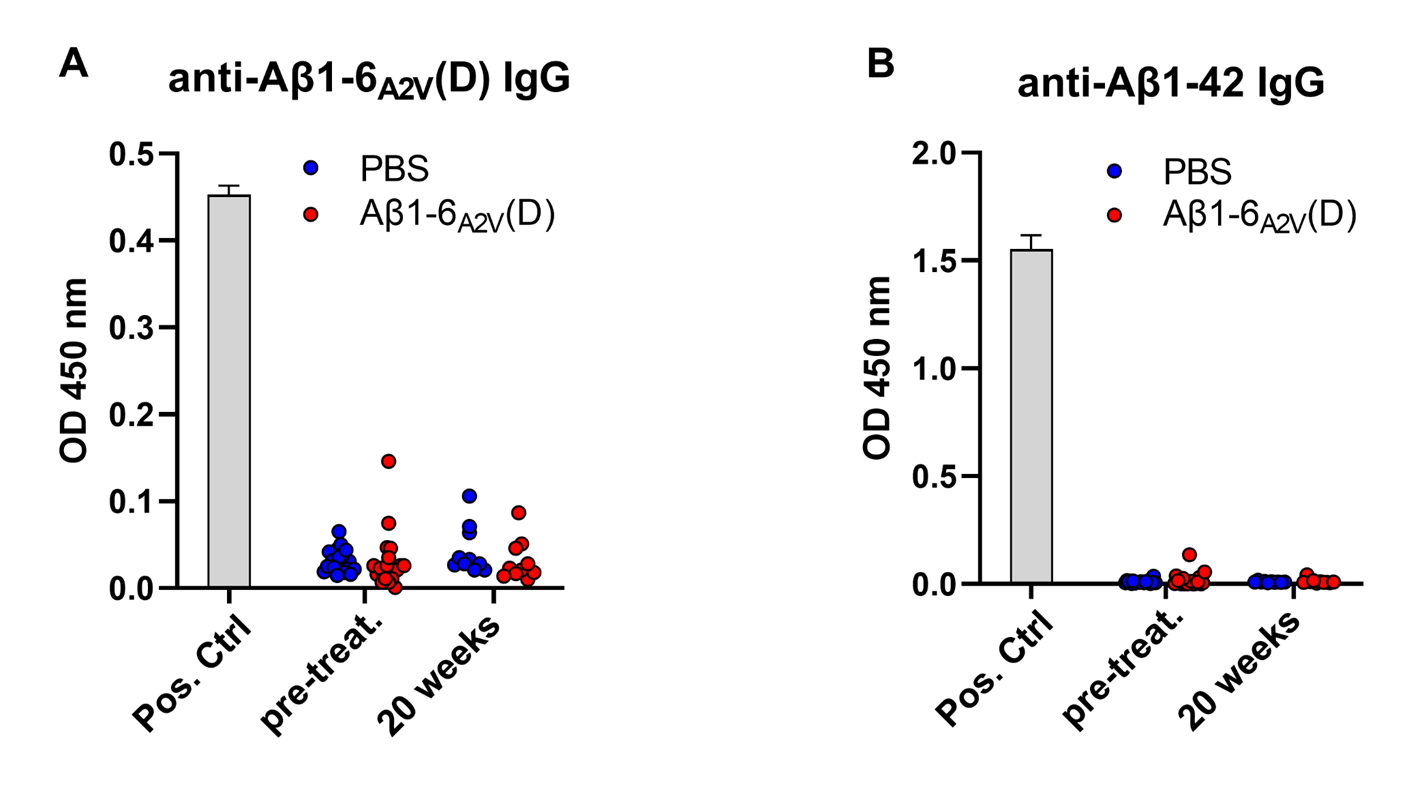


**Supplementary Fig. 3** Sera from mice administered with PBS (blue circles) or Aβ1-6_A2V_(D) (red circles) were collected before starting the treatment (n = 10 each group, pre-treat.) and 20 weeks after the beginning of the treatment (PBS, n = 10; Aβ1-6_A2V_(D), n = 10). Titers of total IgG specific for Aβ1-42 or Aβ1-6_A2V_(D) peptides were tested individually in duplicate by ELISA. Data are expressed as individual values and represent optical density (O.D.) at 450nm. Bars represent the positive controls (pos. ctrl) and are expressed as mean ± SD. No statistical difference between any experimental group was observed using Kruskal-Wallis with Dunn’s multiple comparison tests.
